# "The Hospital" Nursing Mirror

**Published:** 1897-05-15

**Authors:** 


"The Hospital" Nursing Mirror.

Being the Nursing Section of "The Hospital."

[Contributions for this Section of "The Hospital" should be addressed to the Editor, The Hospital, 28 & 29, Southampton Street, Strand, London, W.C., and should have the word "Nursing" plainly written in left-hand top corner of the envelope.]

### News from the Nursing World.

### THE DUCHESS OF ALBANY.

H.R.H. the Duchess of Albany, who has recently been staying at Southsea, paid a private visit to Haslar Hospital, in order to see the sick and wounded of the recent Benin Expedition on behalf of the Queen. The Duchess, who was conducted through the wards by Admiral Sir Nowell Salmon and Inspector General Turnbull, spoke to each patient, and also visited the pensioners' wards and other parts of the hospital.

### THE DUCHESS OF TECK.

All our readers will have been grieved to hear of the Duchess of Teck's sudden illness, and of the necessity for an operation. Her Royal Highness was suffering from strangulated umbilical hernia. Mr. Herbert Allingham was called in by Dr. Wadd, and a consultation arranged with Mr. Tom Smith, the operation being performed by Mr. Allingham. Matters have gone well, and the daily bulletins from White Lodge state that the Princess "is progressing favourably and gaining strength." The critical time is safely over, and it is expected that Her Royal Highness will soon be well on the road to recovery.

### MISS NIGHTINGALE AND DISTRICT NURSING.

The following letter from Miss Florence Nightingale, dated April 17th, was read at the meeting in aid of the Hammersmith and Fulham District Nursing Home at which Princess Louise was present last week:—"I have been in familiar touch with district nursing ever since its first establishment in Bloomsbury. I do indeed look upon it as one of the most hopeful of the agencies for raising the poor physically and morally, its province being not only nursing the patient, but nursing the *room*, showing the family and the neighbour how to second the nurse; also and eminently how to nurse health as well as disease, and especially the health of infants and young children, a matter of national importance, for it includes feeding (above all feeding) them, clothing, and cleanliness. For if a child sets out in life with digestion weakened, feeble mind, and craving for stimulants, its prospect is poor indeed. It includes being a friend and helper—not a patron or almsgiver—to the poor family, which receives her as a friend to mind and body. And I bid you Godspeed with all my heart and mind."

### THE CHILDREN'S TRIBUTE.

Mrs. Jack Johnson read "The Children's Story of the Queen's Reign" on behalf of "The Children's Salon" collection in aid of the Queen's Jubilee Nurses' Commemoration Fund at the Mansion House last week. A large audience gathered in the Egyptian Hall, presided over by the Lady Mayoress, amongst the ladies on the platform being the Duchess of Somerset, the Countesses of Selborne and Bective, Lady Arthur Hill, and Lady Jeune. A resolution was passed "That the work of the Queen's Nurses in attending the sick poor throughout the country in their own homes, helping them back to health, and teaching them the simple laws which govern health, is a work of the greatest national importance." It was also resolved to invite all English-speaking children to take part in the movement. Contributions to the fund were received by the Lady Mayoress.

### NURSING AT ST. GEORGE'S HOSPITAL.

Now that the new Nurses' Home, which is known as the "Major Charles Hall Memorial Home for Nurses of St. George's Hospital," is in occupation, all the wards at that hospital are available for the use of patients, those that were closed having been now re-opened. This addition to the number of beds has involved an increase in the nursing staff, in order that the arrangements made some time since for allowing daily hours off duty for all the nurses might not be interfered with. Ten nurses have therefore been added to the staff, and it is proposed to lessen the consequent increased expenditure by the introduction of a certain number of paying probationers.

### DISTRICT NURSING IN CARLISLE.

The Carlisle District Nursing Association has just held its annual meeting, at which a heavy winter's work was reported. The Association has had three nurses busy through the winter, and in recording their steady, persevering work the Committee add their conviction that the "loving ministry" of the nurses is much appreciated by grateful patients. Dr. Barnes, who presided at the meeting, in an interesting speech, drew a comparison between nursing in the present day and 30 years ago, and told his audience that in his student days at Edinburgh one nurse was considered sufficient for 36 patients. The Association needs support, expenses during the past year having exceeded receipts by some £30.

### NURSING AT LAMBETH.

At a recent meeting of the Lambeth Guardians a very satisfactory report was brought forward of the manner in which the probationers had passed their examination. Of the thirteen probationers who came up for examination nearly all passed with credit, and three with distinction. The Rev. W. Hobbs suggested that the Board should take some steps to show their appreciation of the good result, and the matter was finally referred to the Workhouse and Infirmary Committee for consideration.

### NORTH-EASTERN HOSPITAL FOR CHILDREN.

The committee of the North-Eastern Hospital for Children are appealing for £15,000 to enable the building to be completed according to the original design, and for increased donations and subscriptions. This hospital is unknown to many people, hidden away as it is in a region where its help is sorely needed, but seen only of those whose business takes them to its very doors. In the midst of a crowded neighbourhood, Hackney Road, Shoreditch, where children simply swarm, it yet has only 57 beds, accommodation quite unequal to the demands of the district. Pitiful are the cases that have to remain out-patients for want of room, and it is computed that at least two more or less urgent cases are refused admission every day. There is also not enough space for the adequate accommodation of the nursing staff, some of whom have to be quartered in a house near by at considerable cost and inconvenience. A kind friend has promised £500 towards the building fund provided £7,000 is raised before the close of this year, and it behoves every friend of this excellent little hospital to do what they may to help towards this most desirable end.

### TRAINING FOR MIDWIVES.

On Monday, May 3rd, Mrs. Hewer gave the first of her course of lectures on Monthly Nursing at the Midwives' Institute, 12, Buckingham Street, and her pupils might well congratulate themselves on the Council's choice of a teacher. Mrs. Hewer, who is a trained nurse and a midwife, and the author of "Antiseptics for Nurses" and "Our Baby," gives her instruction in a most clear and helpful manner, dealing minutely with every detail of the nurse's duties from the day she accepts an engagement to its termination. There will be nine of these classes on Mondays at three o'clock, and these are followed on the same afternoon by lectures from the Institute's Medical Instructor, F. R. Humphreys, Esq., L.R.C.P., illustrated by numerous diagrams and models. Nurses who may wish to attend any special lecture separately can do so for a small payment.

### FAREWELL TO MISS CARVOSSO.

Before leaving the Derbyshire Royal Infirmary the matron, Miss Carvosso, whose resignation on account of ill-health is a cause of much regret to all connected with the institution, entertained the Infirmary Saturday Committee to tea in the board-room. The committee took advantage of this opportunity to present Miss Carvosso with a handsome gold bracelet as a memento of her four years of office. Mr. Woodward, the chairman, who made the presentation, thanked Miss Carvosso for her past kindness to the members of the committee, and for her ever-ready sympathy. Universal regret was expressed at her departure, and many cordial wishes for her future welfare. Miss Carvosso received also a number of other pretty and useful presents from the nursing staff and the servants.

### NURSES FOR GREECE.

Four more nurses left London on Wednesday for Athens, sent by the *Daily Chronicle* Fund. They are: Miss Alice Mary Winder, trained and certificated at the London Hospital, late night superintendent at the General Infirmary, Bedford; Miss Jessy S. Parson, trained and certificated at King's College Hospital; Miss Margaret Moody, trained and certificated at the London Hospital, late night superintendent at the Seamen's Hospital, Greenwich; and Miss Kathleen Waller, trained and certificated at University College Hospital. We understand that Mrs. Ormiston Chant has returned to London. Is it possible that there really were no wounded Christians to need nursing?

### NURSING IN DURBAN.

The last few months have seen an abnormal amount of sickness in Durban, and during this period nurses have been much in request, and the number available by no means adequate to the needs of the public. A nurse writing to us from there the other day tells us she has been engaged continuously since last September, and has work in prospect, with but a few days' intermission, up to the autumn. So great has been the demand for nurses that an instance was related in the *Natal Mercury* where application was made "to no fewer than thirty-five nurses" before one could be found at liberty to undertake the case. Now Durban is returning to its usual healthy condition. South Africa generally would appear to have experienced an exceptionally unhealthy season.

### A TREAT FOR MANCHESTER NURSES.

At the invitation of Mr. and Mrs. Laverton, of Redclyffe, Victoria Park, Manchester, the matrons and a number of the sisters and nurses of the Southern, Maternity, and Cancer hospitals, spent last week a most pleasant day at Redclyffe. A band played during the afternoon and evening, and the visitors played games, and wandered about the grounds to their hearts' content. Afterwards they were entertained to dinner, and at the close of of an enjoyable evening were driven back to their respective hospitals. The kindness and hospitality of the host and hostess will be long remembered; and Mr. and Mrs. Laverton are to be congratulated on the success of their kindly plan to give pleasure to their nurse guests.

### A WEDDING.

Old "Londoners," and others who have taken an interest in the work of the Mission to Deep Sea Fishermen on the coast of Labrador, will be much interested to hear of Nurse Ada Carwardine's marriage to Dr. W H. Graham Aspland, which took place in New York, on April 9th. Both have been workers for the Mission, Miss Carwardine for several years past, and she and her husband are returning to take up the respective posts of matron and medical officer at the Battle Harbour Hospital.

### SHORT ITEMS.

The Princess of Wales has consented to receive purses at the garden fête which is to be held in the grounds of the Chelsea Royal Hospital in July, in aid of the Homes for Nurses of Soldiers and Sailors' Families.—A number of nurses from Denmark and Sweden have gone to Greece in order to offer their services on behalf of the sick and wounded.—After opening the Victorian Exhibition at the Crystal Palace last week, Princess Christian presented warrants to sixty-three officers and eighteen superintendent nurses of the St. John Ambulance Brigade.—A Birmingham medical woman, Miss Caroline Green, has been elected to the post of medical officer at Lincoln County Asylum by twelve votes against seven given to a male candidate. —It has been decided to affiliate the St. Austell Nursing Association with the County Association. Mr. Layland-Brratt, High Sheriff of Cornwall, has offered to place at the disposal of the committee a new house in Tregarne Terrace as the head-quarters of the association.—At Kirkcaldy a proposal to commemorate the Queen's reign by the erection of a Victoria Nurses' Home, and its partial endowment, has been unanimously adopted at a well-attended public meeting. A free site has been offered by Mr. Nairn.—It is proposed to establish a district nurse at Wolverton.—A committee of ladies at Holbeck are getting up a fund to present the District Nurses' Institution with a bicycle for the use of the nurses.

### Lectures to Surgical Nurses.

By H. A. LATIMER, M.D. (Dunelm), M.R.C.S. of Eng., L.S.A. of London, Consulting Surgeon, Swansea Hospital; President of the Swansea Medical Society; Lecturer and Examiner of the St. John Ambulance Association, &c.

### VII.—BURNS AND SCALDS. — SPASM OF THE GLOTTIS.—TREATMENT OF THESE AFFECTIONS.

RESUMING the consideration of burns and scalds, on which we were engaged at the close of the last lecture, I now point out that when the heat has been slight, or, being of a severe kind, has only been applied for a very short time, inflammation of the skin only, marked by more or less redness, due to the injection of the fine blood vessels, which occurs in inflammation will arise. If the burning or scalding body has been more intense, or has clung more severely to the parts, the skin will be raised into blisters, and then we have what is known as "vesication." You have all seen blisters, and I should like to explain to you what they are. You remember that they consist of bladders on the skin, more or less large, according to the degree of irritation which has produced them; when pricked a watery fluid escapes from them. This watery fluid is serum derived from the blood. It has escaped from the blood vessels during the process of inflammation, and lies beneath the outer layer of the skin, and upon the deeper and sensitive layers. If the destruction by the application of these agents is again more complete, the true skin, which lies beneath the outer layer, the sensitive part of that tissue, and which contains the endings of the nerves, is killed, and with it we may have more or less death of parts beneath, such as muscles, tendons, and even bones.

Now the most painful of these three degrees of burns and scalds are the first two, because here we have the nerve endings in the true skin, which had not been destroyed, irritated and inflamed, and so, from the point of view of pain, the condition which is most serious is the least painful; but it stands to reason that the shock to the system is likely to be more severe in these cases, where the true skin and the deeper seated parts have been destroyed. What are the dangers then? They are local, or to the part itself; and remote, or to the system at large. Little danger is likely to arise in the lesser degrees of burns—they heal kindly with proper remedies, and do not leave scars; but where the true skin has been destroyed, the healing is necessarily complicated by scar tissue, for, wherever true skin has died, or been removed, it can only be replaced in this manner. The peculiar danger which follows the graver burns is the contraction which is prone to take place in the scars after the wounds have been healed. For this reason you will have to be very careful in the dressings which are applied to these graver burns, and you cannot be too particular in seeing that the splints and restraining apparatus which are put on by the surgeon during the process of healing are kept in place. It is customary in the case, say, of a limb which has been burnt or scalded in this grave manner, to apply splints whilst it is extended or stretched out as far as it is capable of, so that during the process of healing the scar may be as big a one as possible; for we know that that scar will contract later on, and it follows that the larger it is the less the limb will be drawn up and distorted when that stage of contraction sets in. For the lesser degree of burns, where redness of the skin, for instance, is all that has taken place, Carron oil—a mixture of equal parts of lime water and linseed oil—or some other suitable application, is applied on lint, and, should vesication have occurred, the blisters are pricked to allow of the escape of the fluid within them, and again suitable remedies are applied. Be very careful, however, never to cut blisters; the scarf-skin of which they are composed acts as a shield and protection to the tender, deeper, sensitive skin, and hence it is very important that it should not be separated or torn from the part which it covers. The one great thing to be borne in mind in the treatment of these burns is to exclude air: and this is the reason why so many different remedies find favour—cotton wool, flour, linseed oil, and rags, being applied indiscriminately with this object. When you see a case which has been dressed before it has reached you, be very careful whilst removing the dressings that you do not tear the blisters.

Now for the succeeding dangers. The first one is the thing which may occur immediately. You will expect fainting or shock to come on at once, and when these conditions are passed off, to have a degree of reaction. Burns and scalds which have occurred about the head, chest, and abdomen, are dangerous from the especial liability of inflammations of parts which lie beneath them. In the head, for instance, there is liability to erysipelas; and we may have an inflammation of the membranes which cover the brain itself, a condition which is known as "meningitis." In burns of the chest we are liable to have pleurisy, or pericarditis—inflammations, that is, of the serous membranes which enclose the lungs and heart respectively; and inflammation of the lungs themselves may take place. When the abdomen has been burnt, that great serous membrane, "the peritoneum," which lies within it, may become inflamed; and in all extensive burns we are liable to have blood poisoning, from absorption of matter from the wounds and the burnt surfaces. And there is one especial danger which is run, in the shape of ulceration of the bowels. Hence it follows that burns are very grave injuries, and that from the point of view of general treatment, they require watching closely, and long-continued attention.

One especial form of scald I must draw your attention to before I pass from the subject. Young children, when left alone to play in rooms where a kettle is on the hob, seem to be very fond of drinking out of its spout, and so it happens that we not rarely see cases of scalds about the mouth and throat. Now where this has occurred, although the little sufferer may appear to be breathing fairly easily on admission to the ward of the hospital, you must bear in mind that he is liable to a condition of the extremest gravity setting in at any moment. All the air that gets into our chest gains access to the same through a very narrow aperture called "the chink of the glottis." This aperture is contained in the part which everyone knows as "Adam's apple"; it is a triangular opening, which is at all times very small. When the windpipe, in which this aperture lies, is inflamed from any cause, a sudden spasm is apt to occur from time to time, the muscles contracting suddenly and closing the opening. This is known as "spasm of the glottis." When it occurs the patient is immediately thrown into a state of the very greatest danger; he struggles for breath, the face becomes livid, the eyes are staring, frantic efforts are made to breathe, and, in the case of children especially, general convulsions are apt to take place. Spasm of the glottis is particularly liable to be set up after scalds about the mouth and throat, and measures have to be adopted for preventing it, and you have to be aware of the likelihood of its occurrence. In the nursing of these cases it is customary to put the child in a tent bed, so that he may be shielded from draughts of air, which are liable to excite spasm, and the bronchitis kettle is made to conduct steam within the tent, because moist air is much less irritating to the breathing organs than is dry air. Should such a condition as the one I have described set in in a patient whom you are nursing, you will have to be most prompt in sending for the surgeon, who will probably perform the operation of tracheotomy, an operation in which an opening is made into the windpipe below the constricted point so that air may reach the lungs by a new channel.

### Post-Graduate Clinics for Nurses.

By a Trained Nurse.

### XIII.—SOME SAVING GRACES.

Since writing my last sermonette on the domestic economy of illness, a friend whose husband suffered recently from an attack of pneumonia showed me the resultant bill from the chemist for drugs, &c. Among other somewhat extravagant items and lavish quantities figured "eighty-five pounds of crushed linseed." Now it must be confessed that linseed is not a ruinously expensive commodity, and a certain amount of latitude may be given in its use in the sick-room; but the chemist in question "billed" the linseed at elevenpence a pound, and at this sum the total works out at a considerable figure when the amount used approaches a "century."

Considering the short poulticing period usual in a case of pneumonia, it is quite safe to hazard the obvious conclusion that at least half of that linseed was left adhering to poultice bowls and spatulæ. And it is just towards this kind of extravagance that I wish to direct attention and to quote the following as showing the logical results of a want of domestic training in modern home life, and, further, to accentuate the fact that this deficiency is by no means remedied by three years in a Hospital Training School.

I was discussing this very question recently with the progressive Matron of an important London hospital, and she quite concurred with my views. "The more I see of probationers," she said, "the more I blame the modern mother for the insufficient and unsatisfactory domestic training she gives her daughters. To judge from the ignorance of the simplest housewifely details on the part of the average probationer who comes here for training, I look very hopelessly on the future homes of England. And it is a great hardship to a Matron who has so much that is technical to teach the nurse, to have to begin at the beginning and instruct probationers in the elements of housewifery. Only a few days since I received a most striking object lesson in proof of the necessity that every candidate before entering hospital should give some evidence of possessing the average accomplishments belonging to our sex. The hospital sempstress was taken suddenly ill, and there was some work on hand it was absolutely necessary should be finished at once. So I requisitioned the services of one of the nursing staff to step into the gap, and do duty for a short time in the sewing-room, till I could get a substitute for the sempstress. But, throughout the hospital—which contains about 175 beds—there was not a single nurse or probationer who could work a treadle machine! So I had to undertake the duty myself, and as I treadled the machine and ran over the seams—work which I could ill spare the time to do—I accompanied the rhythm of the machine with a veritable hymn of gratitude and praise towards my mother, who brought me up and trained me as a practical, domesticated woman. Then again," she continued, "how easily one sees from the method of cutting dressings adopted by the new probationer that her experience of scissors and 'cutting out' has been distinctly limited, while in nine cases out of ten the gauzes she wastes in fashioning 'dressings,' and the inefficiency displayed by her in cutting out pneumonia jackets and other invalid necessaries, shows an elementary knowledge of sewing that one hardly expects to meet with out of a Board school. I do most heartily agree with you that an essential requirement in the trained nurse is that she should have a certain skill in cutting out and sewing. Quite apart from the practical value to her of this art in helping her to pad splints and make the greatest number of dressings out of the smallest amount of material, dressmaking and sewing gives a deftness of finger and a skill in manipulation which is of the utmost use in bandaging and in handling the sick. I feel so strongly on this subject that I am contemplating introducing this new qualification of domestic knowledge and skill into regulations affecting the admission of candidates to my Training School."

At the Homœopathic Hospital the domestic training of the nursing staff is admirable, and it would be difficult to find in many hospitals such exquisite examples of skill and beauty in the dressings and in the lovely embroideries on blankets, counterpanes, and tray-cloths—all of which is the work of the nursing staff—used throughout this charming hospital. Opening any drawer at random in the children's ward here will at once reveal the most lovely baby and child *trousseaus* made entirely by the nurses. Here one can find charming wool bootikins, knitted by the nurses, so that no small feet may be cold; while dainty bibs, lace-trimmed and embroidered, protect clean gowns from the ravages, made by trickles from careless little mouths, of milk and "baby-food." And all these things are done in spare moments by the nurses, whose great delight is to use their skilful fingers to beautify their patients and their wards. A nurse trained here can hardly help acquiring the daintiest neatness in her use of the needle—a knowledge which will prove of inestimable value be she aiming at a Matronship, or if she intend to take up private nursing, to say nothing of the admirable wife a nurse so trained may make to some fortunate and admiring husband!

Speaking from a long experience, it appears to me that no woman makes a truly good nurse unless she naturally possess some modicum of the housewifely instinct—an instinct which needs perfection by practice, habit, and training. Were I to recount and to set forth in their entirety some instances out of my own experiences of the total absence of housekeeperliness and domesticity in new probationers, I fear I might be accused of setting down a good deal in malice—or a suspicion might arise as to whether such things can be. Some day I intend to collect all the interesting samples I have come across in a book of "Some Pros. I Have Met"; but that, as Rudyard Kipling says, will be "another story." But it may be amusing to recall an occasion on which I detected a fresh recruit to the nursing ranks of a ward in which I was "staff" in the act of filling a foot-warmer and using a stethoscope as a funnel. I must confess here that the stethoscope was by no means an ineffectual weapon to choose for this novel purpose. My reflections on the hopelessness of her up-bringing were framed rather in sorrow than in anger, and, going back to first causes, I was inclined to hold her mother responsible for not having taught her the methods of funnels and the philosophy of kitchen utensils. On another occasion a probationer was caught red-handed in the futile attempt to cut bread and butter with a poultice spatula. She said "she thought it was some new-fangled kind of bread-knife, and she was sure she had seen the cook at home using one for some purpose or other." The incident was so funny we forgot to lecture her on her hopeless unpracticality, for she did not display so much ingenuity in her invention of machinery as did the probationer who used a stethoscope as substitute for a funnel. That same probationer remains to this day an awful example among "nurse failures," having gone about from hospital to hospital vainly trying to find a permanent resting-place. But each in turn refuses to harbour her within their walls, and she bids fair to become the Wandering Jew of the nursing profession. No sooner does she pitch her tent in some position or other than she receives notice to quit, for she has carried her domestic unpracticality into her profession, and Matrons will have "none of her."

### A Question of Age.

By a Matron.

What is the best age to begin to train as a nurse?

Women enter the nursing world either of necessity or from choice. Necessity makes many seek employment at the earliest possible opportunity as a means of subsistence, but in the present remarks I refer rather to those whose lives are more or less influenced by inclination.

Few hospitals of any repute accept candidates under twenty. Most of our children's hospitals recruit their probationers between the ages of twenty and twenty-five, whilst the large general hospitals receive them from twenty-five to thirty-five. For our present purpose we will presume that most educated gentlewomen enter the nursing profession with the hope of eventually becoming the matron of a large hospital. For this they must be fully qualified, especially in these days of keen competition. They must have received a thorough hospital training, they must be competent to undertake the control and instruction of the nursing staff, and must have a practical knowledge of household management, and they must not be more than forty years of age. To acquire such qualifications must take at least seven or eight years. In order to be fully competent to enter upon the responsibilities of the matron's office, it is well to have had practical experience in the various branches which will come under her superintendence. The aspirant must not have forgotten the details of probationers' work; she should have learned to nurse on day and night duty under the surveillance of the ward sister; she should thoroughly understand ward management as sister; she should be accustomed to teach probationers and nurses both practically and theoretically; and, finally, she must become acquainted with household management, either as assistant matron or matron in a small institution. Then she will be prepared to climb to the top of the ladder. Seven or eight years is none too long a time to study this curriculum. Hence it is better not to postpone the commencement of the training after the age of thirty.

The question then arises, how *early* is it advisable to begin? Personally, I should not recommend anyone to make their start until they are twenty-seven or twenty-eight years old. The glow of youth has then been satisfied, the "fever and the fret" of early life run less high, and are replaced by a clearer mind and calmer judgment, "a more unselfish love, a more unclouded truth." The maturer nature is better able to bear the shock which intimate acquaintance with disease and accident and their manifold causes must necessarily awaken. For practical reasons, also, it is a mistake to begin too soon. Nurses who have entered a children's hospital at twenty find themselves at the end of their two years' training still too young to receive their general training in most of the large hospitals. Their first energy has gone, and they have to face the disheartening fact that until they are older they may find it difficult to ensure employment.

To my mind twenty-eight or twenty-nine is the ideal age for those who can choose; I am well aware that there are many who cannot afford to wait so long, and who at an earlier age are forced to face the problem of existence. To such I would say in the words of Miss Nightingale, "Leave these jargons, and go your way straight to God's work in simplicity and singleness of heart."

### Minor Appointments.

Cork Street Fever Hospital, Dublin.—Miss Mary L. Watson has been appointed Assistant-Matron at this hospital. Miss Watson was trained at the Baggot Street Hospital, Dublin, where she has since had the charge of the fever wards.

West Kent Hospital, Maidstone.—Mrs. Chester has been appointed Night Sister at this hospital. Mrs. Chester was trained at the New Hospital for Women, Euston Road, and at the West Ham Hospital.

### A Lady Army Surgeon.

Surgeon-Major-General Gore, writing to *The Lancet* recently, gave a most interesting extract from the second volume of "Fifty Years of My Life" by the Earl of Albemarle concerning the late Dr. Barry, Inspector-General of Hospitals. Lord Albemarle stopped at the Cape, *en route* from the Mauritius to England, and writes as follows:—

"There was at this time at the Cape a person whose eccentricities attracted universal attention—Dr. James Barry, staff surgeon to the garrison, and the Governor's medical adviser. Lord Charles described him to me as the most skilful of physicians and the most wayward of men. He had lately been in professional attendance upon the Governor, who was somewhat fanciful about his health; but the Esculapius, taking umbrage at something said or done, had left his patient to prescribe for himself. I had heard so much of this capricious yet privileged gentleman that I had a great curiosity to see him. I shortly afterwards sat next him at dinner at one of the regimental messes. In this learned Pundit I beheld a beardless lad, apparently of my own age, with an unmistakably Scotch type of countenance—reddish hair, high cheek-bones. There was a certain effeminacy in his manner which he seemed to be always striving to overcome. His style of conversation was greatly superior to that one usually heard at a mess-table in those days of non-competitive examination.

"A mystery attached to Barry's whole professional career, which extended over more than half a century. While at the Cape he fought a duel, and was considered to be of a most quarrelsome disposition. He was frequently guilty of flagrant breaches of discipline, and on more than one occasion was sent home under arrest, but somehow or other his offences were always condoned at headquarters.

"In Hart's Annual Army List for the year 1865 the name of James Barry, M.D., stands at the head of the list of Inspectors-General of Hospitals. In the July of that same year the *Times* one day announced the death of Dr. Barry, and the next day it was officially reported to the Horse Guards that the doctor was a woman. It is singular that neither the landlady of her lodging nor the black servant who had lived with her for years had the slightest suspicion of her sex. The late Mrs. Ward, daughter of Colonel Tidy, from whom I had these particulars, told me further that she believed the doctor to have been the legitimate granddaughter of a Scotch earl, whose name I do not now give, as I am unable to substantiate the correctness of my friend's surmise, and that the *soi-disant* James Barry adopted the medical profession from attachment to an army surgeon who has not been many years dead."

### Where to Go.

Workhouse Infirmary Nursing Association.—The annual gathering of the Mary Adelaide nurses will take place on Tuesday, May 25th, at the Portman Rooms, Baker Street, at eight p.m. A short address will be given by Miss Gibson, matron of the Birmingham Infirmary.

Queen's Hall, Langham Place.—A grand afternoon concert will be held at this hall on Tuesday, June 1st, in aid of the North-Eastern Hospital for Children, Hackney Road, at which a number of well-known artistes have promised to give their services. Tickets £1 1s., 10s. 6d., 5s., and 3s. Admission 2s. 6d. Tickets may be obtained from the secretary of the hospital, Mr. T. Glenton-Kerr, at the City offices, 27, Clement's Lane, E.C., or from any of the usual agents.

Canterbury Cathedral.—On May 31st Sir Henry Irving will read Tennyson's "Becket" in the magnificently restored Chapter House of Canterbury Cathedral, for the benefit of the Thirteenth Centenary fund. There will be an exceptional interest in hearing Sir Henry Irving read Lord Tennyson's work in the midst of the scenes in which the memorable death of the great Archbishop took place. St. Thomas Becket entered the Chapter House on the evening of December 29th, 1170, only a few minutes before his murder in the adjoining "martyrdom." The restored Chapter House will be reopened by H.R.H. the Prince of Wales on Saturday, May 29th, and will be first publicly used for this reading.

### A Nurse's Visit to Texas.

III.

This week I will try to give some idea of what is particularly interesting to us nurses, the condition of the sick and of those who meet with serious accidents in the thinly-settled districts of Texas. Of course, in the large towns there are fully qualified physicians and surgeons, but large towns are few and far between, so in most of the large villages such as Blanco City—every village is a city out there—one or more men will be found who call themselves "doctors," but whose sole claim to the title is that they have read up a little about medicine and surgery and gained some empirical experience at the expense of their patients. While I was out I heard of a butcher giving up his trade and taking to "the profession"; perhaps he thought previous experience would help him in his anatomical studies.

A student from Edinburgh came out on a visit and was persuaded to stay and set up in practice; and though he was not qualified, still he ought to be better than men who had had no hospital experience, but he was said to have no success with broken limbs.

Fortunately there is not much illness in the country, thanks to the pure air and the simple hard-working life all are obliged to lead, just such a one, I should imagine, as would please the Dr. Keith who has lately published his "Plea for a Simpler Life."

Chills and fevers and felons, the latter being very painful boils, were the most frequent troubles, and those who avoided living in a hollow or near the water were rarely, if ever, troubled by the former. The quacks manage these common ailments very well; it is in the event of serious accidents that they fail their patients. They cannot deal with bad gunshot wounds, limbs torn and crushed in the cotton ginning mills, or with the internal injuires sometimes caused by riders being thrown from their horses and dragged. If the patient can afford the expense and the case admits of delay a qualified doctor could be summoned from one of the large towns, but in most cases the poor patient is taken thirty or forty miles over the rough roads before he can get skilled treatment. Sometimes quite hopeful cases will sink from exhaustion and die before reaching their destination for want of stimulant, for Blanco is a prohibition county, and even in cases of the direst necessity alcoholic liquors cannot be bought even at the chemists. I must explain the reason of this. Early settlers in Texas earned for themselves the character of being a very wild lot, scapegraces and fugitives from justice from Europe and the States, and in some parts they are so still, so although the generality are now quite everyday folk, their bad reputation still clings to them, and to prevent mischief the law forbids them to buy spirits or beer, or to carry their shooting irons outside their own pastures, on the supposition that if they get the former they will assuredly use the latter. So much for the liberty of the subject in the "Lone Star State." When a bad accident happens to a Texan, friends of the sufferer will ride round and try to find some English settler who has brandy in stock—as such a man generally has—buying it outside the prohibition boundary and keeping it ready for any emergency.

Properly qualified medical men have certainly no temptation to settle up country; cases are few and far between, pay uncertain and frequently "in kind," not coin, so the local practitioner is obliged to keep a ranch on which to put the cows, &c., which come to him as fees. Texans seem to me to be in a chronic state of "hardupness"; ready money was certa nly very scarce in our locality.

Of course, under these circumstances, trained nurses for the country are out of the question. They would rarely get patients, and could not farm to keep themselves in slack times. When there is sickness in a house, neighbours are exceedingly kind, and always ready to lend a helping hand. In one village the chemist used to undertake the duties of professional night nurse in serious cases.

Had I thought it possible to make a living, I would have set up there myself, for a very few months of that splendid air completely cured my chest symptoms, and my brother, who had active lung mischief when he went out, has felt nothing of it during his seven years' residence.

I have used all the space allowed to me, so must keep the rest of my remarks on these subjects for my next article.

### The Plague at Poona.

Miss Catherine McIntosh who, it will be remembered, went out to Bombay to help in the nursing of the plague patients some two months ago, sacrificing a portion of her furlough from Hong Kong (where she holds the appointment of sister in the Government Civil Hospital) in order to do this, has now been placed in charge of the nursing arrangements at the plague hospital at Poona. Soon after her arrival in India Miss McIntosh was sent to Poona, and here her previous experience of the plague at Hong Kong at once made her services of much value. She at first accompanied the search parties on their house-to-house visitation, women being much needed to undertake this work in the zenanas. The need for skilled nursing amongst these unfortunate patients at Poona has been great indeed. At the hospital, the biggest plague hospital in the Presidency, there are no less than 230 beds. Of these the greater number were occupied by women and children, the sole attendant allotted to each female ward being an untaught ayah, while equally ignorant ward boys were in charge of the male sheds. This state of things could not last, and now, in consequence of urgent representations that trained nursing should be instituted at once, the whole of the arrangements for the nursing at this hospital have been put into Miss McIntosh's hands. A bungalow has been taken not far from the hospital (which is itself but a collection of mud huts in the midst of an immense sandy field), where Miss McIntosh and a staff of nurses have taken up their abode, and are now busily occupied in getting matters into working order. Two sisters of the Indian Nursing Service have joined her, and there is much to be done. Most of the cases of plague are surgical at one part or another of their course, and the dressings alone occupy a great deal of time, to say nothing of the daily routine work. Surgeon-Major Barry is the medical officer in charge of the hospital, the rest of the staff consisting of a Parsee doctor, who is practically "house-surgeon," and several hospital assistants, or native students.

The stringent measures adopted in order to battle with the disease have had excellent effect, and the death-rate in Poona has been steadily diminishing. The story of the house-to-house search parties, the military escort, the ambulances, the men and carts armed with all the paraphernalia for fumigating, lime washing, &c., the prompt separation of the sick and the healthy, the former to hospital, the latter to the huge segregation camp, must be ancient history now to English readers. At Poona these operations have been conducted on a very large scale, a camp of nearly a thousand men having been set apart for this work.

The Jaffray Hospital, Birmingham. — Miss Ethel Godfrey, whose appointment as Sister of the medical floor at this hospital was recently noticed, asks us to add that she received her training at Charing Cross Hospital before going as sister to the Royal Victoria Hospital at Bournemouth.

### Everybody's Opinion.

[Correspondence on all subjects is invited, but we cannot in any way be responsible for the opinions expressed by our correspondents. No communication can be entertained if the name and address of the correspondent is not given, or unless one side of the paper only is written on.]

### DISPENSING.

Miss Bradbury (resident dispenser), Ryde Dispensary, Isle of Wight, writes: On reading the correspondence *re* "Dispensing" in a recent number of The Hospital, I cannot but think it is a mistake to advise a girl to work for the preliminary examination of the Pharmaceutical Society during the time she is supposed to be giving to practical and theoretical pharmacy. In the case of youths about to enter a chemist's business it is a different matter; but surely for ladies it would be better either to get it over before entering a school of pharmacy, or where this is impossible—which is often the case with nurses and those anxious to begin actual dispensing at once—to leave these subjects, which do not bear directly on the work, till after a certificate has been obtained from the Society of Apothecaries to act as an assistant. In this way my pupils are able at the end of their six or eight months' training to earn their own living, and to get with no further expense the three years' practical experience required before passing the minor. It was through reading your advice in "Burdett's Hospital Annual" some five or six years ago that I adopted this plan, and have since found it answer exceedingly well.

### THE ABUSE OF UNIFORM.

"A. L. M." writes: In reply to the letter from "E. F. G." in a recent issue of The Hospital, it may be a help to her to know that I have met with the same "nurse frauds." To protect my nurses I some time ago designed badges for both my private and district nurses to wear outside their cloaks, and these have been much appreciated by the nurses and by the doctors and the general public. In addition to this badge my private nurses when at a case wear an armlet with the initials of the institute worked upon it. The district badge has three colours, red, white, and blue; the private, red and white only. I think every institution or hospital should insist upon its nurses wearing a badge designed especially for their use, and then this abuse of uniform could not be. I should be glad to know the opinion of other superintendents upon this matter. From my own experience this badge expedient has satisfied all parties interested in our nurses. There has been a greater demand for our nurses, and I am sure they are treated with greater respect. I do not think a "Jubilee" badge would be any protection, as it could not possibly guarantee all its wearers to be trained nurses.

### AN OPENING FOR THE PARTIALLY TRAINED.

"One who Desires Incompleteness" writes: I have just read with hilarity a suggestion by a correspondent signing herself "One who Desires Completeness," to the effect that the detrimentals, who, from delicacy or incompetence, are rejected from our hospitals, should band themselves together, and under the ægis of some "club, institution, or office" step in and complete the work of the trained nurse. To quote—"There comes a time when the skill of a trained nurse is no longer necessary." Exactly. But where, then, is the "homely, handy girl," or "unencumbered widow"? Is she not capable of looking after a convalescent relation? In most cases where the family cannot afford a private nurse, the district nurse is to be had to give her help and advice. Surely this is "robbing Peter to pay Paul" with a vengeance. As a certificated nurse I protest against thus sweating the profession, and glutting the maket with inferior stuff! Is there nothing in the world for a woman to do but become a nurse? As well suggest that all the quacks, and men who are sent down and plucked yearly, should offer their services to the public as doctors, parsons, &c., at a lower remuneration. I hardly think that your correspondent's "plan" is likely to "take root and flourish" in the mind of any member of the nursing profession.

### The Book World for Women and Nurses.

[We invite Correspondence, Criticism, Enquiries, and Notes on Books likely to interest Women and Nurses. Address, Editor, The Hospital (Nurses' Book World), 28 & 29, Southampton Street, Strand, London, W.C.]

The Narrative of My Experience as a Volunteer Nurse in the Franco-German War of 1870-1. By Anne Thacher. With a Sketch of her Life by James M. Menzies, M.A. (Abbott, Jones, and Co., Limited, 4, Adam Street, Strand. 92 pp., with portraits Price 3s. 6d.)

This brightly-written narrative is not without a certain pathos and dignity as we follow its heroine from her life in a luxurious English home to the trials and hardships of active service in the garrison hospitals of Cologne. The early bereavement, through death in the battlefield, of her lover, who perished from lack of proper nursing in the terrible winter of the Crimean War, determined Miss Thacher to offer her services in the Franco-German War, and in spite of every obstacle, she succeeded in attaining her object. It is interesting to read of her work under the French sisters in Garrison Lazareta and the little luxuries and indulgences she was able to introduce for the benefit of the patients. Germans and French were treated alike in the military hospitals, and Miss Thacher tells us "she was all for the Germans, yet she felt more sympathy for the French sick as they had no friends to come and visit them." The French were as humane as the Germans, and it is pleasing to learn that "their hearts could be equally touched by the sight of suffering, even though they were the enemies of their country." "I owe my life," said one man, "to French kindness," and spoke of them in touching terms of gratitude. He happened to be a bricklayer and his brother was a cabdriver in Coblentz. Miss Thacher, having qualified herself in every possible way, was at length assigned a tent (No. 30) in the Tent Hospital. There she acted as superintendent, and had two nurses under her. The tent consisted of three wards, containing 24 beds, and Miss Thacher "discharged not only the duty of a nurse, but even that of surgeon to the wounded soldiers when the necessity arose, the regular medical attendants being so overtasked that they gladly left minor operations to one who proved herself so competent to fulfil them."

On conclusion of the war, it is pleasing to read of the various public acknowledgments she received for her self-denying labours, not the least valued of which were the personal compliments paid her by the Empress Frederic, who received her at the German Embassy and expressed her deep regret at being unable to visit the hospital at Cologne. Among the many amiable traits possessed by this lady must be mentioned her love of animals, with one or two charming anecdotes of which this interesting little book is brought to a close.

### Appointments.

### MATRONS.

Borough of Bolton Fever Hospital.—Miss Annie M. Kirsoff has been appointed to fill the post of Matron at this hospital. Miss Kirsoff was trained at the Monsall Fever Hospital, Manchester, subsequently working as night nurse at Newcastle-upon-Tyne, and as charge nurse at the Birkenhead Hospital and the Bolton Borough Hospital.

Invalid Asylum, Stoke Newington.—Miss Amy Coke has been appointed Matron at this institution. Miss Coke received her training at St. Bartholomew's Hospital and at Queen Charlotte's Hospital. She had held the post of matron at the Harlington and Eltham Cottage Hospitals and at the Windsor Infirmary.

### For Reading to the Sick.

"LIGHT."

**Verses.**

LEAD, kindly Light, amid the encircling gloom,
Lead Thou me on;
The night is dark, and I am far from home,
Lead Thou me on.
Keep Thou my feet; I do not ask to see
The distant scene; one step enough for me.

I was not ever thus, nor prayed that Thou
Should'st lead me on;
I loved to choose and see my path; but now
Lead Thou me on.
I loved the garish day, and spite of fears,
Pride ruled my will; remember not past years.

So long Thy power hast blest me, sure it still
Will lead me on
O'er moor and fen, o'er crag and torrent, till
The night is gone,
And with the morn those angel faces smile,
Which I have loved long since and lost awhile.
—*Newman.*

Lord! if our Fathers turned to Thee
With such adoring gaze,
Wondering, frail man Thy Light should see
Without Thy scorching blaze;
Where is our love, and where our hearts—
We who have seen Thy Son—
Have tried Thy Spirit's winning arts,
And yet we are not won?
The Son of God in radiance beamed,
Too bright for us to scan;
But we may face the rays that streamed
From the mild Son of Man. . . .
God, by His Bow, vouchsafes to write
This truth in Heaven above
As every lovely hue is light,
So every grace is love.—*Keble.*

I longed for Light; but all the Light I found was second-hand,
Reflected thought that had been tossed about for ages past,
From surface-minds that vainly claimed alone to understand
The mystery of the Light that is like Shadow on us cast.—*W. Smith.*

**Reading.**

I am the Light of the world; he that followeth Me shall not walk in darkness, but shall have the light of life. —*John* viii. 12.

Once God said, when He created all things by Jesus Christ, "Let there be light." It was the first act of that mighty drama, of which the curtain rose on Chaos and fell on Paradise. . . . We do not always appreciate as we ought this part of our Saviour's office. He is the Light as well as the one Chief Object which the Light has to make visible. He is not the Redeemer only, He is the Revealer too. . . . We have all to make our way through the wilderness of this world to some destination—and what? We cannot answer the question. Sometimes life seems as if it led nowhere; as if it were rather (what Scripture sometimes calls it) a "walk," a journey, a little circuit, bringing us back at night to the home from which we started, rather than an extended progress to determine in an end unlike its beginning. . . . Christ comes to us then as the Light; shows us the Path which we were missing, the outlet which we were ignoring; says to us, "This is the way, walk ye in it," when we should certainly have turned from it to the right hand or to the left.—*C. J. Vaughan.*

### Notes and Queries.

*Nurses for Greece.*

(246) Where or to whom ought I to apply as a candidate for the Cretan expedition? I hear more nurses are going out.—*Urlton.*

The nurses who have gone out to Greece have been sent by the *Daily Chronicle* fund, and application should be made to that by nurses wishing to offer their services. But the chances are, we should imagine, quite against any more nurses being sent out.

*Tracheotomy.*

(247) Please tell me of some work on tracheotomy?—*Theory.*

You will find a nurse's duties in a case of tracheotomy fully dealt with in Dr. Percy Lewis's "Nursing; Its Theory and Practice." (Scientific Press, 28 and 29, Southampton Street, Strand.)

*Training.*

(248) I am anxious to become a probationer when I have reached my twentieth year in the coming autumn, but fear that my present occupation (that of bar-attendant) would prevent my acceptance. I see in "How to Become a Nurse" that there are hospitals which will take probationers at twenty. Please tell me if it will be of any use applying?—*Hopeful.*

If you are suitable for a nurse's training, and can give satisfactory references, there is no reason why you should despair of gaining admission to a hospital or infirmary as probationer. Apply to the matrons of some of the hospitals and poor-law infirmaries mentioned in "How to Become a Nurse."

*Schott Treatment for Heart Disease.*

(249) Please tell me in which numbers of THE HOSPITAL shall I find reference to Dr. Schott's treatment of heart disease? I remember seeing it described.—*St. Leonards.*

You probably mean the articles by Dr. Richard Greene and Dr. Theodore Fisher in the numbers for October 19th, 1895, and January 11th, 1896. You can get these back numbers on application to the manager at the office, 28 and 29, Southampton Street, Strand. The first-named article is also published in pamphlet form by the Scientific Press.

*Nursing Abroad.*

(250) Kindly tell me where to apply for information respecting the nurses going out to Bermuda? If they are not yet selected I should like to offer to go. I have been twice out to South Africa, so am not afraid of distances.—*Miss N.*

Please see rules respecting answers by post. You had better write to Mrs. Piggott, hon. secretary of the Colonial Nursing Institute, Imperial Institute, S.W.

*Army Nursing Service.*

(251) Can you tell me if a nurse who has had three years' training in a good poor law infirmary would be eligible for admission into the Army Nursing Service or the Army Reserve Staff? Where should I apply for information on the subject?—*Infirmary Nurse.*

Apply to the War Office for all information respecting the Army Nursing Service or the Army Nursing Reserve.

*Private Nurses' Travelling Expenses.*

(252) Please tell me what rule should be observed by private nurses not working in connection with an institution as to payment of travelling expenses? Thus if I go from my home in London to nurse Mrs. A. at Manchester, and from there to Mrs. B. at Chatham, thence returning to my home, should I charge Mrs. A. the fare from London to Manchester and from there to Chatham, or should the latter journey be put down to Mrs. B's account? It seems difficult to settle the matter fairly to both parties except by always charging the return fare from home.—*Justitia.*

The rule of the Nurses' Co-operation with regard to travelling expenses says: "A nurse must only charge travelling expenses to and from the office to her case." A private nurse is entitled to charge her travelling expenses to and from her home in each case. If it happens that she finds it more convenient to go straight from one patient to another without returning home, she must be guided by circumstances as to any reduction a consequent saving of expense may induce her to think it right or desirable to make. She must not in any case charge any one patient a higher sum for travelling expenses than would be incurred by a direct journey from and to her headquarters, except if, at the patient's own stipulation, she has for any special reason to take a more costly route.

*Age for Training.*

(253) Please tell me at what age probationers are first admitted into any London crêche.—*Dora.*

There are some institutions for children at which probationers are admitted from 18 or 19 years of age. Read "How to Become a Nurse" (Scientific Press, 28 and 29, Southampton Street, Strand).

*Appointment Wanted.*

(254) Can you tell me how to obtain an appointment as assistant matron or lady superintendent of a nursing institution, not having a nurse's training, and being 39 years of age? I would not mind giving six months' services or paying small premium.—*Reader.*

We should recommend your giving up any idea of making application for such a responsible post. It is fortunately becoming increasingly rare to appoint any lady to the important office of superintendent of a nursing institution who is not herself a trained nurse. You should try for some appointment which does not require special training and qualifications.

*Advertisement.*

(255) Can you tell me in what part of England the provincial hospital is situated which is advertised in THE HOSPITAL for May 1st?—*Southampton.*

You must write to the advertiser yourself. We cannot give private information respecting advertisements appearing in our columns.

